# Prospective comparative clinical trials of novel non-invasive intracranial pressure pulse wave monitoring technologies: preliminary clinical data

**DOI:** 10.1098/rsfs.2024.0027

**Published:** 2024-12-06

**Authors:** Vilma Putnynaite, Edvinas Chaleckas, Mantas Deimantavicius, Laimonas Bartusis, Yasin Hamarat, Vytautas Petkus, Andrius Karaliunas, Arminas Ragauskas

**Affiliations:** ^1^ Health Telematics Science Institute, Kaunas University of Technology, K. Donelaicio street 73, Kaunas LT-44249, Lithuania

**Keywords:** non-invasive measurement, intracranial pressure, intracranial compliance, intracranial pulse waves

## Abstract

Intracranial pressure (ICP) monitoring is crucial in the management of traumatic brain injury (TBI) and other neurological conditions. Elevated ICP or too low intracranial compliance (ICC) can compromise brain perfusion. Simultaneous monitoring of ICP and ICC is needed to optimize patient-specific brain perfusion in pathological conditions. Surrogate ICC changes can be extracted by analysis of ICP pulse wave morphology. Non-invasive, fully passive sensor and ICC changes monitoring are needed. This study introduces Archimedes, a novel, fully passive, non-invasive ICP wave monitor that utilizes mechanical pulsatile movement of the eyeball to assess ICP pulse waveforms. Preliminary findings indicate a high correlation *r* = [0.919; 0.96] between non-invasive and invasive ICP pulse wave morphologies, demonstrating the device’s potential for accurate ICP pulse waveform monitoring. Additionally, the monitor can discern ICC changes, providing valuable insights for TBI and normal tension glaucoma patients according to the shape of non-invasive measured ICP pulse wave. The k-nearest neighbours algorithm used in preliminary glaucoma studies yielded promising diagnostic performance, with an accuracy of 0.89, sensitivity of 0.82, specificity of 1.0 and area under curve 0.91. Ethical approvals for ongoing studies have been secured. Initial results indicate that Archimedes real-time ICC non-invasive monitor is safe, cost-effective alternative to conventional monitoring techniques.

## 1. Introduction

Brain neurons can be damaged or die when intracranial pressure (ICP) rises above the patient-specific limit, and intracranial compliance (ICC) decreases below the patient-specific toleration limit. Optimal cerebral perfusion pressure (CPP)-guided therapy was introduced as a concept, which allows personalization of CPP management to improve the outcomes for patients with traumatic brain injury (TBI) [1].

Marmarou *et al*. [2] first described ICC as the ratio of intracranial volume change to change in ICP (ICC = ∆Volume/∆ICP). Brain swelling, disturbed venous outflow after TBI or increased cerebrospinal fluid (CSF) volume in hydrocephalus cases are the leading causes of decreased compliance. However, too high compliance was associated with normal tension glaucoma compared to healthy subjects [3].

The scientific community agrees that ‘ICP is more than a number’, and the morphology of the ICP pulse waveform gives more diagnostic information than the ICP value [4]. The physiological ICP pulse waveform has three expressed morphological peaks: P1, P2 and P3 [5]. By elevating the mean ICP value or decreasing ICC, the ICP pulse waves become progressively rounded, where the peaks disappear. The peak ratio of P2/P1 is associated with ICC value [6–8]. If the P2/P1 ratio is above 1, it is associated with abnormally low ICC due to different brain pathologies of intracranial volume compensation capacity [9]. The amplitude of the ICP pulse wave is directly related to the mean ICP value [10]. During the appearance of intracranial slow waves, the amplitude and morphology of the ICP pulse wave have higher variability compared to cases of absent slow waves [11]. Recent studies have shown a similarity in shape between ICP and cerebral blood flow velocity waveform during ICP plateau waves [12]. The studies [13–15] showed that the P2/P1 ratio or the amplitude of the ICP pulse wave (whether measured invasively or non-invasively) is the significant marker for outcome prediction after TBI.

In cases such as cardiac surgery, organ transplantation, ophthalmology and aerospace medicine, where invasive ICP sensors cannot be implanted into patients’ brains, there is a need for non-invasive technologies capable of accurately and precisely monitoring ICP pulse waveforms. Several studies show that MRI, rheoencephalography (REG), photoplethysmography (PPG) or optical coherence tomography (OCT) technologies have the potential to estimate compliance-related ICP pulse wave characteristics non-invasively. However, the clinical application of these technologies is still limited.

A study using the REG method based on measurement of electrical impedance showed REG waves with three morphological peaks. However, the bioimpedance variable cannot extract ICP pulse wave morphology because it also reflects scalp blood volume pulsation [16]. The study conducted using the PPG approach of brain pulse monitoring showed a correlation coefficient of *R* = 0.66 between non-invasive PPG pulse waves and invasive ICP pulse waves in TBI patients [17]. ‘brain4care’ (Brazil) developed a novel non-invasive monitor B4C that detects small skull pulsation due to ICP changes and pulse waves. The correlation coefficient between P2/P1 ratios measured with invasive ICP and B4C monitor was equal to 0.41 [18].

The biophysics of intracraniospinal CSF volume and ICP pulsatility are well understood [19–23]. CSF volume and ICP pulsatility in the subarachnoid space around the optic nerve have also been under investigation in recent years [24]. It has been shown using OCT that the optic nerve head pulsates with an amplitude of 7.8 ± 1.3 µm [25]. Optic nerve head pulsation is a cause of an eyeball pulsatile movement. In a pilot study [26], it was shown that there is no correlation between intracocular pressure and the pulsatile optic nerve head displacement amplitude. The OCT parameters of the peripapillary Bruch’s membrane angle (pBA) and maximum optic nerve head height (ONHH) also correlate with overnight pulsatile ICP. The pBA became increasingly smaller with increasing pulsatile ICP; however, ONHH increased with increasing ICP pulsatility [27].

According to the results of state-of-the-art analysis, the general requirements for a non-invasive monitor of ICP pulse waves are as follows:

— a sensor of such monitor has to be passive: no transmission of some physical signals (electrical, electromagnetic such as radio waves or NIRS and ultrasonic) to the human head, no pressure applied to the human eye,
— no indirect, nonlinear or correlation-based relationship between invasively recorded ICP pulse wave shape and non-invasively recorded pulse wave shape has to be a basic principle of operation of non-invasive ICP pulse wave and ICC change monitor and its sensor,
— no distortions of non-invasively recorded ICP pulse wave shape by blood volume pulsations in the human scalp,
— clinically acceptable accuracy and precision of non-invasive spatially averaged ‘global’ (not sectoral or local) ICP pulse wave shape monitoring in both hemispheres of a human brain to identify ICC changes in cases of TBI, stroke, hydrocephalus, glaucoma, sport and aerospace medicine, etc.

We hypothesize that using the phenomenon of eyeball mechanical movement, which directly follows ICP pulsations, it is possible to create a novel, non-invasive sensor and a wireless, real-time monitor of ICP pulse waves according to the general requirements listed above.

This article presents the preliminary clinical data of two prospective comparative clinical trials to validate a novel non-invasive ICP pulse wave monitoring technology.

## 2. Methods

### 2.1. Archimedes—non-invasive intracranial pressure pulse wave monitor

**Figure 1.**
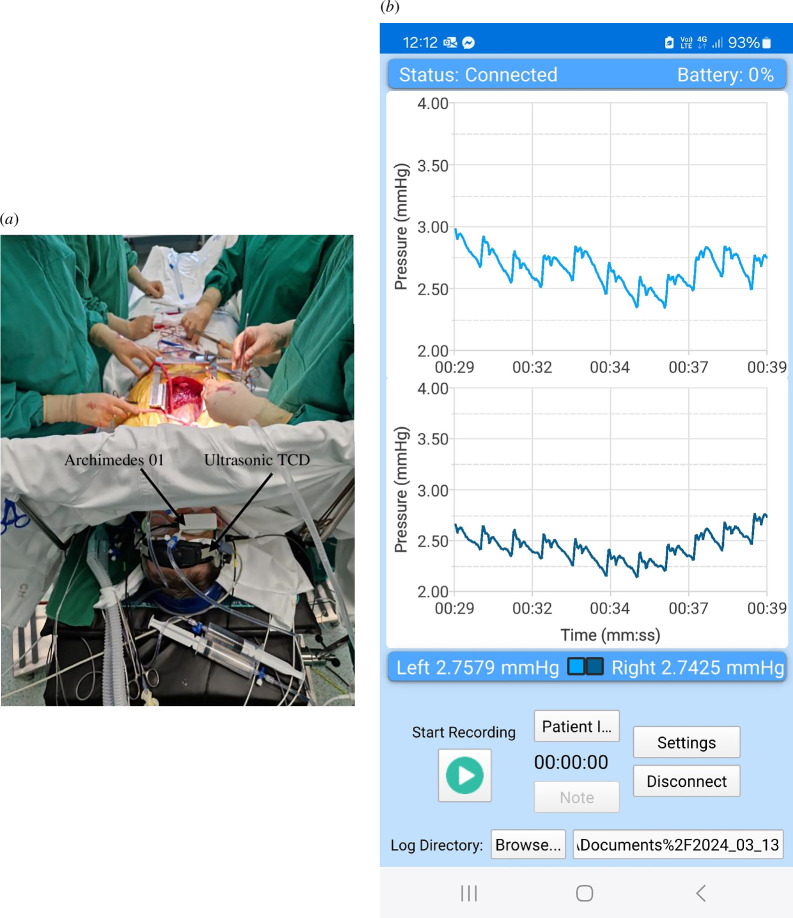
Archimedes 01 sensor placed on a patient during cardiac bypass surgery together with transcranial Doppler (Dolphin 4D, Israel) head frame (*a*), and the smartphone screen that presents non-invasive ICP pulse waves recorded from the left and right eyes (*b*).

**Figure 2.**
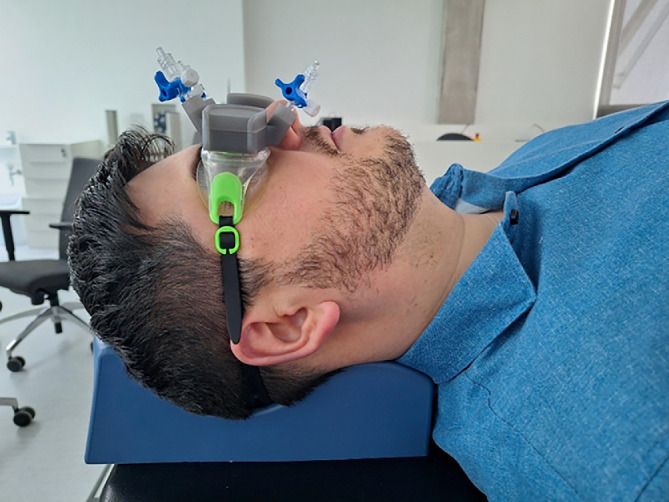
Non-invasive wireless ICP pulse wave monitor Archimedes 02 placed on a healthy volunteer.

Following our ideas and formulated requirements above, we created a novel non-invasive, fully passive ICP pulse wave monitor Archimedes 01/02. It is based on ICP pulse wave sensing by monitoring the mechanical pulsatile movement of the eyeball (figure 1*a*) through a closed eyelid. The device consists of one (Archimedes 01) or two ‘goggles’ (Archimedes 02) attached to the closed eyelids of the patient. The internal medium of the ‘goggles’ is isolated from the closed eyelids with a thin (50 μm) non-allergic elastic film. The inner volume of the goggles is filled with a non-compressible liquid, and a digital pressure sensor is placed in it. The principle of operation is based on the fact that cerebrospinal fluid pulsation in the subarachnoid space of the optic nerve causes an associated spatial movement of the eyeball. The pressure sensor of a wireless Archimedes real-time ICP monitor is connected to a medical microelectronic Bluetooth transmitter of pulse wave signals, which transmits real-time ICP pulse wave signals to a laptop or smartphone. Presentation on the screen of the smartphone of pulse waves recorded from the left and right eyes of a healthy volunteer is shown in figure 1*b*. The monitor’s ICP pulse wave sensor is lightweight and completely passive, i.e. it does not radiate any physical signals to the eye, orbit or intracranial medium and does not add any pressure to the eye or orbit. The ‘goggles’ are cost-effective and disposable. The upgraded version of the Archimedes 02 device (figure 2) can monitor ICP pulse waves through both eyes simultaneously in order to diagnose a difference in ICC changes in injured and healthy brain hemispheres after TBI, stroke, intracranial vasospasm or re-growing brain tumour.

### 2.2. Ethical approvals

The ethical approvals were obtained to implement two ongoing prospective comparative observational studies (2024–2025) for clinical validation of Archimedes technology: ‘R&D of non-invasive innovative intracranial waves monitoring system for diagnostics and treatment monitoring of patients with normal tension glaucoma’ (project no.: SV5−40) and ‘Research and development of innovative non-invasive high temporal resolution ICP waves monitoring technology’ (project no.: S-MIP−23–68). Clinical data collection is carried out in accordance with the study protocols approved by the Vilnius (Lithuania) Regional Biomedical Research Ethics Committee (nos. 2024/3−1570−1030, 2024−03−05) for brain injury patients with implanted ICP sensors and by Kaunas (Lithuania) Regional Biomedical Research Ethics Committee (no. BE−2–15, 2024−02−10) for normal tension glaucoma patients comparing with a control group of healthy volunteers.

Participants and/or their legal guardians signed a written informed consent form to participate in the study. The anonymized clinical data are used for analysis. The study was registered at the clinical trial registration system ClinicalTrials.gov. The study identifier is NCT06443411.

### 2.3. Data collection and analysis

For ICU patients, non-invasive ICP data (recorded with Archimedes 01/02) and invasive ICP data measured with the invasive ICP monitor Raumedic Neurovent-PTO or with external ventricular drainage (EVD) and ICP monitoring system were collected using ICM+software (Cambridge, UK). The sampling frequency of the recorded data was 100 Hz. The duration of data monitoring sessions was at least 3 min. The monitoring sessions were processed by extracting, detrending and normalizing each pulse wave in the time domain and calculating one averaged pulse wave per monitoring session. Ten ICU patients were included in the ongoing comparative study until now. All patients required routine implantation of an invasive ICP sensor or EVD: six patients with subarachnoid haemorrhage (SAH), two patients with TBI and two patients after brain tumour removal. The patient age range was 31–78 years, and the mean age was 56 years. There were five female and five male patients. The Pearson correlation coefficient was used to check the similarity between invasive and non-invasive pulse wave monitoring of ICU patients. The correlation coefficient was determined between averaged invasive and non-invasive pulse wave monitoring sessions for each patient and expressed by the mean value and interquartile ranges of the overall ICU patients group.

For glaucoma patients or healthy volunteers, non-invasive ICP data only were collected. Currently, 12 healthy volunteers and 43 glaucoma patients’ data were included in the analysis. The age range of glaucoma patients was 43–87 years, the average age was 66 years, 13 male and 30 female patients. The age range of healthy volunteers was 27–33 years, average age was 30 years, with four male and eight female volunteers. The K-nearest neighbours (kNN) algorithm was utilized to develop an artificial intelligence (AI) model for glaucoma diagnosis based on the waveform of non-invasive ICP pulse waves and evaluate the waveform variances in ICP pulse waves among glaucoma patients and healthy volunteers. A dataset from 12 healthy volunteers was used to create an average reference normal waveform, while data from 43 glaucoma patients were averaged to form a reference pathological waveform. These reference waveforms were used to train a kNN algorithm to classify pulse waves as either normal or pathological. The classification was based on the nearest neighbour, determined using the Euclidean distance between the input waveform and the reference waveforms. The receiver operating characteristic (ROC) analysis was performed to estimate the kNN model characteristics (accuracy, sensitivity, specificity and an area under the curve) for diagnosing glaucoma.

## 3. Preliminary results

**Figure 3.**
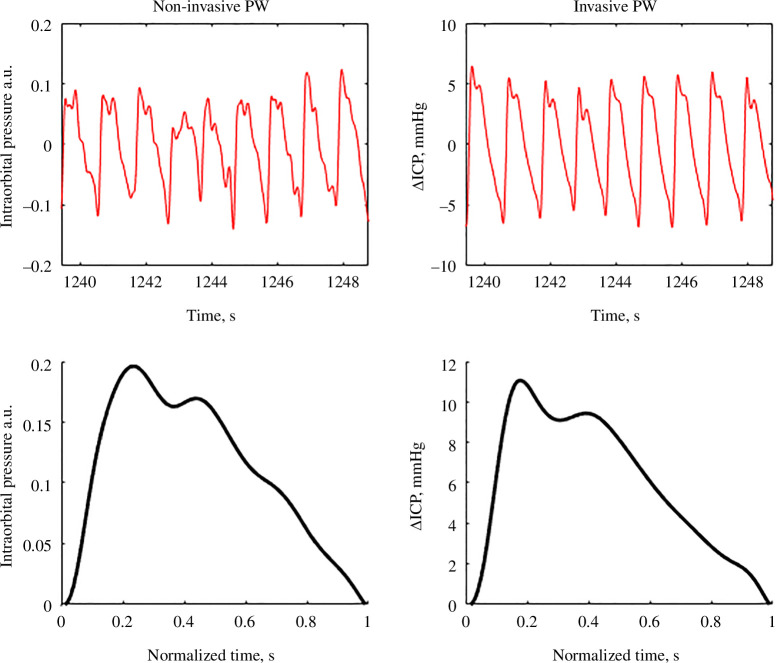
The comparison of non-invasive (*a*) and invasive ICP pulse waves (*b*). The raw monitored signals of simultaneously recorded non-invasive and invasive ICP pulse waves are shown in red curves (top graphs). The processed non-invasive and invasive ICP pulse waves, after eliminating slow and respiratory waves, normalization, and averaging, are shown in black curves (bottom graphs). The invasive ICP pulse waves were recorded using the Raumedic Neurovent-PTO invasive ICP sensor implanted in the brain parenchyma. The correlation coefficient between averaged invasive and non-invasive pulse waves was *r* = 0.993.

Invasive and non-invasive ICP measurements were performed on patients after SAH surgery, ruptured intracranial aneurysm and intracranial meningioma removal surgery until now. ICP pulse waves were recorded simultaneously, both invasively and non-invasively, for up to 3 min to obtain an average pulse wave shape for each patient. The interquartile range of correlation coefficient between invasive and non-invasive pulse waves for the first 10 ICU patients was from 0.919 to 0.96, with a mean value of 0.933 and standard deviation of 0.063. An example of estimating the similarity of invasive and non-invasive ICP pulse waves is shown in figure 3, where the averaged correlation coefficient per monitoring session is *r* = 0.993. To our knowledge, such a high correlation has never been demonstrated with other non-invasive monitoring technologies that apply NIRS, ultrasound, electrical or radio signals.

In an ongoing clinical study on normal tension glaucoma patients, the monitoring data from non-invasive ICP were collected on 43 patients and 12 healthy volunteers until now. A kNN algorithm trained on healthy volunteers and normal tension glaucoma patients’ waveform data were used to create a model for glaucoma diagnosis. The preliminary results obtained from the ROC of the developed model (accuracy of 0.89, sensitivity of 0.82, specificity of 1.0 and area under curve 0.91) showed clinically acceptable performance for diagnosing an early stage of normal tension glaucoma and monitoring of treatment’s effectiveness. The correlation coefficient between the average reference pulse wave of healthy volunteers and the set of glaucoma patients’ pulse waves was 0.66, with a standard deviation of 0.107 and an interquartile range from 0.6145 to 0.6701.

The pulse wave repeatability analysis was performed on each healthy volunteer to test the non-invasively recorded variations of ICP pulse wave signals on different days. The calculated correlation coefficients between measurements performed on the first day and those on the second, third and fourth days were *R* = 0.997, *R* = 0.917 and *R* = 0.924 for the left eye and *R* = 0.999, *R* = 0.948 and *R* = 0.953 for the right eye, respectively, showing acceptable repeatability of non-invasively measured ICP pulse waves on healthy volunteers.

## 4. Discussion

The guidelines for TBI management emphasize the importance of absolute ICP value monitoring for acute TBI patients by pointing to the evidence-based averaged ICP threshold value, which is associated with unfavourable outcomes [28]. However, many studies show that such critical ICP threshold varies among patient groups and, therefore, does not reflect patient-specific cerebrospinal compensatory reserve [6,8,9,11,29,30]. The ICC changes and its related metrics based on ICP pulse wave morphology were introduced to estimate cerebrospinal compensatory reserve changes, which could open new perspectives for the identification of personal ICP thresholds and challenges against the concept of evidence-based medicine, which proposes a universal and fixed ICP threshold [8,9,30]. In addition, such methodology allows the estimation of ICC changes using non-invasive approaches.

Therefore, our study aims to explore the proposed non-invasive ICP pulse wave measurement technology by analysing the similarity between the shape of invasive and non-invasive ICP pulse waves measured through the mechanical eyeball pulsation caused by the ICP pulse waves.

### 4.1. Limitations

The novel technology proposed for non-invasive ICP pulse wave monitoring is based on our hypothesis that human eyeball pulsation reflects the dynamics of CSF volume and provides the same morphology as the invasive ICP pulse wave. The background for this hypothesis is the anatomical feature of the optic nerve, which is surrounded by CSF and is connected to intracranial CSF [31,32]. Therefore, the pressure in the subarachnoid space, which is equal to ICP, is transferred into the eyeball through the optic nerve head. ICP pulsations can be sensed by eyeball movements with an externally applied pressure sensor, which has hydrostatic contact with the pulsating eyeballs. Our previous studies on the piglet model confirmed that approach [33]. Our ongoing study is the first pilot study of the proposed technology. The first limitation is the limited number of included patients. Additional limiting factors, such as blood flow pulsations in the extracranial skin layers surrounding the eyeball or anatomical abnormalities in the eye orbit, might influence the shape of non-invasively recorded eyeball pulsations. However, the performed comparison of invasively and non-invasively measured ICP waves shows a high correlation between these waves, varying in range from 0.919 to 0.96 with a mean value of 0.933.

Although in this ongoing study with a limited number of patients we used the correlation coefficient as the metric for comparing normal and pathological pulse wave in glaucoma patients, the further comparative analysis will include the nonlinear dimensional reduction technique—Isomap projection of the pulses into bidimensional space [8] and other metrics (ratio P2/P1, time to peak [6,7]) to perform detailed comparison of pulse wave morphology.

Although many studies demonstrate that the ICP pulse waveform can be used to estimate individual patient-specific ICC changes, we analysed the similarity between invasive and non-invasive ICP pulse wave shapes in this comparative study. Larger studies, including long-term ICP pulse wave invasive and non-invasive monitoring over plateau phases, are needed to explore the feasibility of individual patient-specific management of the ICP value and brain compliance management.

We found in our prospective clinical studies [3,34,35] of patients with diagnosed normal tension and high tension glaucoma that in normal tension glaucoma cases an ICP is lower than normal. A consequence of that phenomenon is a deformation of the lamina cribrosa caused by two pressure gradients: normal intraocular pressure and abnormally low ICP. Too low ICP is associated with too high ICC. Our hypothesis of the ongoing normal tension glaucoma study is that Archimedes can be used for normal tension glaucoma diagnosing and treatment monitoring because of the possibility of monitoring abnormally high ICC changes. A key limitation of our ongoing normal tension glaucoma study is the non-invasive character of indirect ICC identification. Unfortunately, direct invasive ICC measurement is impossible in patients with glaucoma because of ethical reasons.

## 5. Conclusions

The novel Archimedes fully passive, non-invasive ICP pulse wave monitor has been developed using, for the first time, direct monitoring of an eyeball mechanical movement caused by ICP pulse waves. It shows a high correlation with simultaneously invasively recorded ICP pulse waves according to preliminary data of a prospective comparative observational study. Also, it showed clinically acceptable repeatability, sensitivity, specificity and accuracy according to preliminary data of a clinical comparative observational study of normal tension glaucoma patients and healthy volunteers. The proposed monitor is safe for the patients. It does not radiate any physical signals to the eye, orbit or intracranial medium and does not add any pressure to the eye or orbit. It is disposable and cost-effective compared with invasive ICP sensors and monitors, and it is wireless and easy to use. It can be used in a wide range of clinical medicine areas inside and outside intensive care units or surgery theatres. It can be used everywhere where ICC monitoring by automatic AI-based analysis of recorded pulse wave morphology is needed for patients treatment decision-making.

## Data Availability

Due to privacy concerns and ethical considerations, access to the clinical data used in this study is restricted. Data are available upon reasonable request, subject to approval by the Regional Kaunas Biomedical Research Ethics Committee (kaunorbtek@lsmuni.lt) and Regional Vilnius Biomedical Research Ethics Committee (rbtek@mf.vu.lt).
